# From Fungicide to Phagosome: A One Health View of Copper-Driven Fungal–Bacterial Interactions

**DOI:** 10.1007/s40588-026-00277-0

**Published:** 2026-07-31

**Authors:** Seána Duggan

**Affiliations:** The Medical Research Council Centre for Medical Mycology at the University of Exeter, Streatham Drive, Exeter, EX4 4QD UK

**Keywords:** Fungal-bacterial interaction, One Health, Copper, Candida, Staphylococcus

## Abstract

**Purpose of Review:**

Copper links environmental, animal and host-associated niches as a micronutrient, antimicrobial, biocide and selective pressure. This review examines how copper exposure shapes fungal–bacterial communities and proposes the “cuprosphere” as a One Health framework for these connected copper-influenced environments.

**Recent Findings:**

Copper inputs from crop protection, livestock feed, manure, wastewater, built environments and host immunity can suppress sensitive microbes, select copper-tolerant taxa, enrich resistance determinants and alter interkingdom networks. In *Candida albicans–Staphylococcus aureus* biofilms, complementary copper-handling responses suggest that copper homeostasis can support mixed-community resilience.

**Summary:**

Copper should be viewed as a One Health ecological force that shapes fungal–bacterial communities across environmental, animal and host-associated niches.

## Introduction

Copper is unusual among antimicrobial pressures because it is simultaneously an essential micronutrient, a cofactor for key enzymes, a host immune defence weapon, an agricultural biocide and an environmental contaminant. Across fungi and bacteria, copper adaptation extends beyond resistance: microbes must acquire copper when it is scarce, detoxify or export it when it is excessive, and remodel their metabolism as copper availability changes across niches. At the individual level, this is mediated by importers and exporters, copper-sensing transcription factors, metallothioneins, multicopper oxidases, copper chaperones and cuproenzymes. In pathogens, those systems are often directly tied to virulence. Copper provides a One Health lens for understanding how environmental pressures shape microbial communities across natural and built environments. Here, I refer to the expanse of copper’s influence as the **cuprosphere**: the ecological continuum across which copper acts as a nutrient, toxin, antimicrobial and selective force shaping microbial survival and interactions.

## Copper as an Organising Principle in One Health

One Health is the integrated and unifying approach linking the health of humans, animals, plants, and their ecosystems. Within this, antimicrobial resistance (AMR) is an exemplar concept as a worsening global problem, with the potential to be best addressed by a One Health approach [1]. Copper stands out within this framework because it is intrinsically cross-sectoral: essential for microbes, animals, and humans, and naturally occurring as a trace metal in soil, water, and air [2]. It is anthropogenically added to crops, livestock diets, medical devices and antimicrobial therapies, as well as retained in topsoils, manures, slurries, hospital effluent and pipe corrosion products [3–6]. During infection, immune cells deploy copper to phagolysomes in concentrations toxic to engulfed microbes [7]. The same physicochemical properties that make copper useful as a fungicide or antimicrobial also make it a persistent selective pressure when it is repeatedly present at non-lethal concentrations. This duality establishes a conceptual bridge of copper as a pressure point from fungicide to phagosome.

The way organisms experience copper exposure should not be considered a single biological “condition”, as all copper exposures are not equivalent. Soluble copper salts in feed or spray programmes, organically complexed copper, persistent soil-bound copper, particulate corrosion products, copper nanoparticles, and metallic copper impose different stresses. Across plants, microbes and animals, copper toxicity occurs when bioavailable copper exceeds homeostatic capacity. Copper toxicity is therefore a conserved failure of metal homeostasis, expressed across living systems as oxidative damage, metabolic disruption, impaired growth or virulence control, organ injury and cell death [8–10]. Sublethal or chronic copper exposures may select for copper tolerance or resistance and promote the persistence of resistance determinants, whereas high-concentration or contact-killing exposures may reduce viable microbial reservoirs and thereby limit their dissemination. This review proposes that copper is an important pressure-point for microbes in the environment and a driver of interactions with their communities and hosts Table 1.

**Table 1:** Glossary of terms

Term	Definition
cuprosphere	The connected network of copper-influenced environments
copper speciation	The chemical form of copper, including its oxidation state, complexation, solubility and particulate form, which determines its biological activity
resistome	The complete set of resistance determinants present in a microbial community or niche
co-selection	Selection for one resistance trait because it is genetically or ecologically linked to another, for example copper resistance maintaining antibiotic resistance
Shannon diversity	A diversity index that accounts for both the number of taxa present and their relative abundance
viticulture	The cultivation of grapevines
rhizosphere	The soil region influenced by plant roots and their associated microbial communities
phyllosphere	The above-ground surfaces of plants, that provide a habitat for microorganisms
biofilm	A structured microbial community attached to a surface or to other cells, embedded within a self-produced matrix
MRSA	Methicillin-resistant Staphylococcus aureus
phagolysosome	The intracellular compartment where engulfed microbes or particles are degraded and killed
calprotectin	A host metal-binding protein that restricts microbial access to metals such as copper during inflammation

### The One Health Copper Cycle

**Fig. 1:**
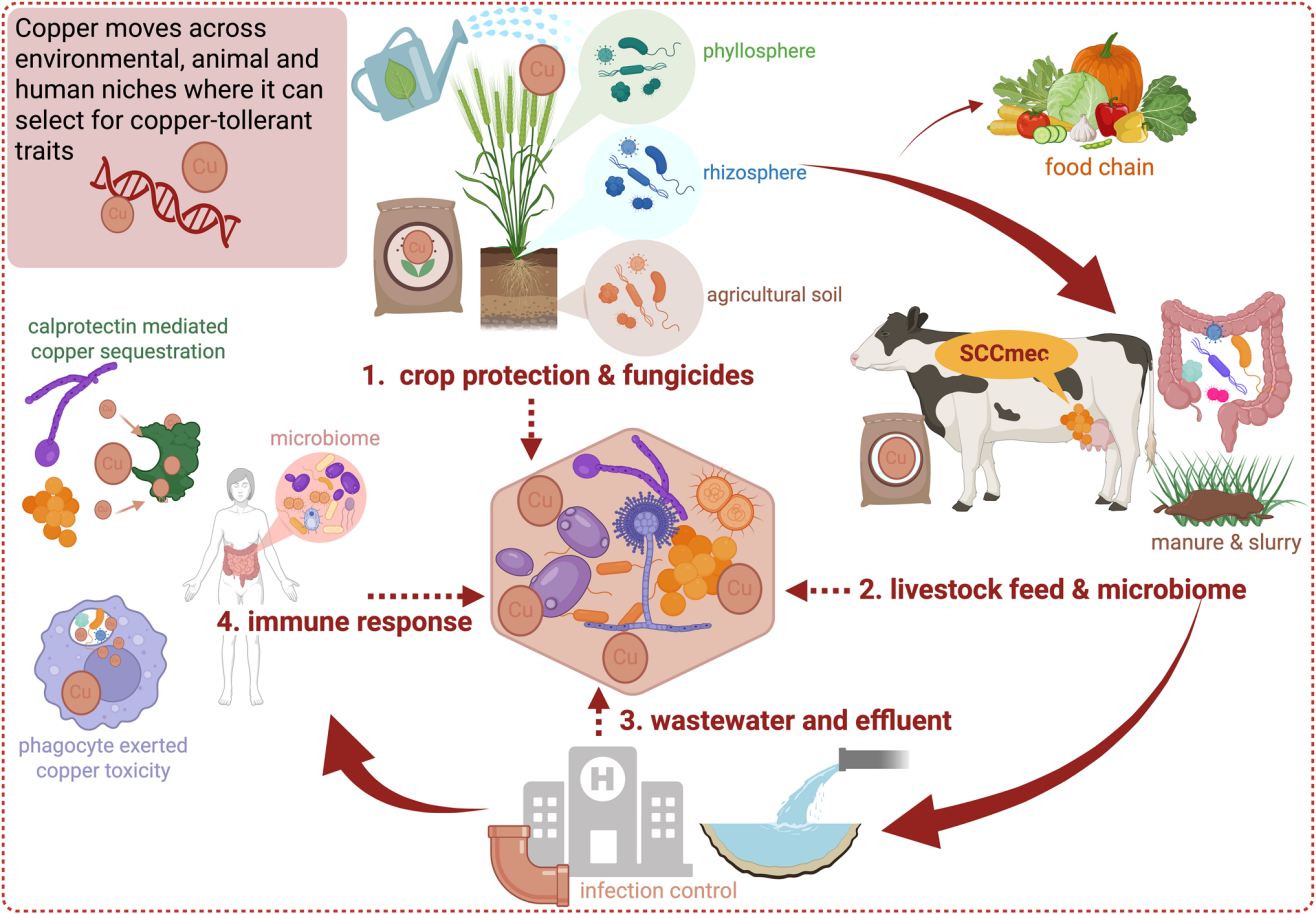
The Cuprosphere: One Health copper pressure points. Copper use in crop protection, livestock feed, wastewater systems and host immunity creates connected selection pressures that may enrich copper-tolerant microbial traits across the food chain, microbiome and infection environments

As a ubiquitous, essential and conditionally toxic element, visualising copper’s roles in a One Health system is best achieved as a cycle rather than a linear exposure chain. Omnipresent at basal levels, copper additionally enters the system through crop protection, animal feed and the built environment such as plumbing and surface technologies. It then accumulates in soils, manures, slurries and effluents where it reshapes microbial communities and resistance networks; reaches humans and animals as both environmental exposure and antimicrobial intervention; is redeployed by the host during infection; and returns to the environment via excreta, wastewater and solids. The opportunity now is to define where along this cycle copper effectively suppresses microbial transmission, and where it instead creates selective conditions that favour more persistent or harder-to-treat consortia Fig. 1.

## Copper Fungicides as Drivers of Non-Target Microbial Selection

Copper-based antimicrobial compounds remain important in horticulture, agriculture and viticulture to prevent fungal disease [11]. They are recognised antimicrobial pressures that can correlate with resistance in plant pathogens [12, 13]. Importantly, prokaryotes generally exhibit lower copper tolerance than eukaryotes [14, 15], meaning copper fungicides are unlikely to act exclusively on fungal targets. Consistent with this, copper exposure has been shown to reduce total bacterial biomass while increasing Shannon diversity, suggesting selection for a copper-tolerant community rather than the intended microbial suppression [16]. Thus, copper biocides prevent fungal disease, but also impose broader ecological perturbations on local microbiomes. Work examining soil, rhizosphere and fine-root microbiomes of tea plants following copper treatment found niche-specific effects, with bacterial and fungal diversity generally increasing in roots and soil, but decreasing in the rhizosphere [17]. Metagenomic analyses identified enrichment of metal-resistance genes, including copper-related determinants such as P-type ATPases, copper-associated genes and copper homeostasis pathways. Copper treatment also enriched fungal taxa with pathogenic potential, including *Botrytis cinerea* and *Fusarium oxysporum*, while reshaping bacterial communities, suggesting that fungal outcomes may be influenced indirectly by copper-tolerant competitors or partners. Notably, leaf raking intensified the copper-associated rhizosphere disruption, highlighting how land-management practices can modulate these outcomes [17]. Exposure time is another important feature, with long-term Cu-metal contamination reshaping bacterial communities and intensified bacterial–fungal co-occurrence networks [18]. These findings indicate environmental copper exposure can indirectly influence fungal persistence through interkingdom ecological restructuring.

In grapevine phyllospheres, copper treatment influenced bacterial community structure more clearly than fungal community structure, while fungal perturbations were driven largely by season, demonstrating how copper effects on mixed leaf microbiomes are filtered through temporal and ecological contexts [19]. Copper hydroxide treatment of tobacco leaves illustrates that copper biocides can remodel the phyllosphere as a mixed fungal–bacterial habitat, reducing some target-associated fungi while also suppressing beneficial bacteria and allowing other potentially pathogenic fungi to expand [20]. Altogether, these findings show copper sprays can suppress target fungi while also restructuring the bacterial species sharing their niche.

## From Feed to Waste: Copper Selection Across Animal and Environmental Reservoirs

In livestock systems, copper is added to pig diets at pharmacological levels that exceed nutritional requirements to improve growth and gut health; however, these inputs also reshape the intestinal microbiota [21], and because much ingested copper is excreted, may extend copper-selective pressure from animal production into agricultural environments. Thus, copper use in food-animal production is an important One Health consideration. High-copper feeding in weaned piglets offers a clear example of how copper can reshape an animal-associated microbiome [22]. While this approach is used to support growth and gut health after weaning, it can also alter the intestinal microbial community by reducing copper-sensitive commensals and favouring more copper-tolerant microbes, which may give copper-resistant pathogens an advantage. In the case of zoonotic *Salmonella Typhimurium* ST34, copper resistance may help the lineage persist in the pig gut and remain within the food-production chain [22].

Evidence linking copper supplementation to AMR co-selection remains mixed and context dependent. In a controlled pig trial, 250 ppm dietary copper increased faecal copper, but did not provide evidence for co-selection of antibiotic-resistance genes or mobile genetic elements [23]. By contrast, a separate study found that 120 mg/kg CuSO₄ expanded antibiotic-resistance and metal-resistance genes, promoted mobile elements, and linked these changes to oxidative stress, barrier dysfunction and beneficial short-chain fatty acid depletion [24]; Interestingly, the more bioavailable copper-peptide formulation had a lower resistome impact than standard CuSO₄ [24]. Altogether, it is likely any copper effect depends on formulation, dose, animal gut ecology and whether exposure is strongly microbiocidal or merely selective.

Transmission pathways also extend beyond the animal itself, with manure, slurry and soil systems acting as important routes for resistome movement. Heavy-metal pollution in soil can reshape microbial communities and co-select for antibiotic resistance, meaning contaminated soils may act as environmental reservoirs of AMR [25]. In a swine manure–slurry–soil system, copper was one of the most abundant metals detected. Here, anaerobic digestion reduced metal concentrations, but increased AMR genes, and their association with mobile genetic elements suggesting co-selection can contribute to the resistome [26]. However, it’s important to note this study supports a broad metal–AMR relationship rather than a copper-specific causal mechanism. Nevertheless, these findings suggest that copper should be considered alongside antibiotics in manure-to-soil AMR surveillance.

Drinking water systems provide an analogous urban reservoir where copper released from pipe corrosion acts as an ecological pressure impacting bacterial growth and increasing resistance genes in lab-scale microcosms [27]. These observations support a One Health model in which copper-enriched wastes and infrastructures act as environmental bridges between agricultural and clinical resistomes.

## Human-Associated Microbes in the Cuprosphere

**Fig. 2:**
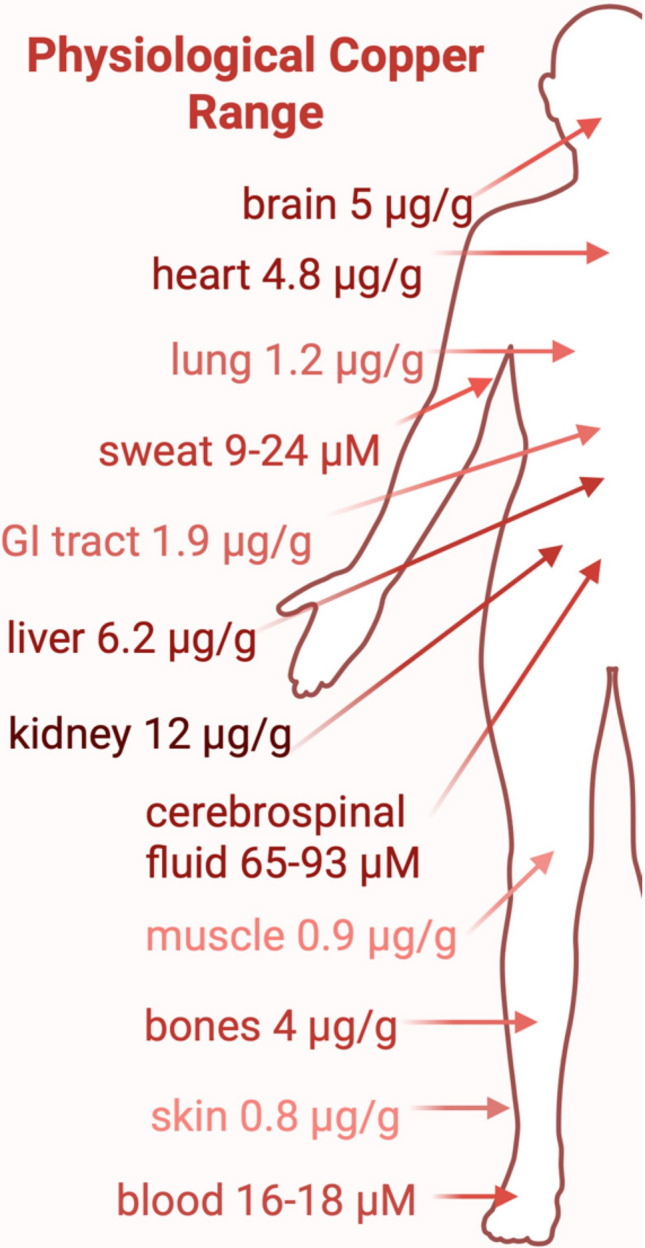
Physiological copper concentrations across human niches. Approximate copper concentrations are shown for selected human tissues and fluids, reported as µg/g for organs and tissues and µM for fluids. The values highlight the niche-specific copper landscapes encountered by microbes across the host, ranging from low-copper environments such as skin to copper-enriched compartments including kidney and blood as described by [38]

As copper enters and moves through the One Health system, what does this mean for microbes that affect human health? Having defined the cuprosphere at the systems level, this review next examines how human-associated microbes respond to copper within it, using *Candida albicans* and *Staphylococcus aureus* as an exemplar interkingdom pair. Both are commensals, opportunistic pathogens, biofilm formers, and World Health Organisation priority pathogens [28, 29]. These organisms are frequently co-isolated from diverse polymicrobial infections at sites such as skin, oral niche, diseased lung [30–34], and systemic coinfection where mortality rates can exceed 50% [35]. Interestingly, these organisms have vastly different tolerances for copper; *C. albicans* tolerates above 1 mM [36], while the *S. aureus* minimum inhibitory concentration is 200 μM [37]. Despite this, both organisms navigate the broad range of physiological concentrations of their human host (Fig. 2) [38].

Our team recently identified a previously unrecognised but critical role for copper in the interaction between these two organisms [39]. This makes them a powerful model for connecting systems-level copper exposures to infection biology: both encounter copper as an environmental pressure, a host-imposed stress, and, in mixed biofilms, a potential mediator of interkingdom interactions.

### Host Copper Landscapes

As commensals and opportunistic pathogens, *C. albicans* and *S. aureus* are exposed to a spectrum of copper concentrations throughout the human host niches they inhabit (Fig. 2). During *C. albicans* infection, mice kidneys initially increase copper concentration up to 24 h, followed by prolonged copper depletion, while in the blood there is an initial increase which is sustained throughout a 96-h infection [40]. Simultaneously, professional phagocytes build mitochondrial copper stores to become activated [41] and concomitantly weaponise intracellular copper by trafficking it into phagosomes through ATP7A-dependent mechanisms, exposing invading pathogens to toxic copper concentrations as part of antimicrobial killing [42]. Additionally, during infection, calprotectin is produced by immune cells and functions to sequester Cu(II), blocking fungal copper acquisition from serum and inducing a copper-starvation response involving copper-saving measures such as remodelling of superoxide dismutase (Sod) activity [43]. This positions calprotectin as an early host mechanism that forces *Candida* to adapt to local copper limitation in addition to broader systemic copper toxicity. In a separate study, the response of *S. aureus* to calprotectin was shown to be a metal-responsive regulatory network remodelling which functions to maintain bacterial fitness and pathogenicity under calprotectin-imposed metal limitation [44]. A mixed *Candida–Staphylococcus* consortium capable of surviving both copper excess and copper paucity would therefore be well suited to the fluctuating metal landscape of host infection [39]. Several mechanistic possibilities exist: fungal uptake and storage may buffer local copper availability, bacterial export and detoxification may reduce copper toxicity, and biofilm architecture may spatially compartmentalise copper to flatten otherwise lethal extremes.

### Copper & *C. albicans*: Flexibility and Virulence

**Fig. 3:**
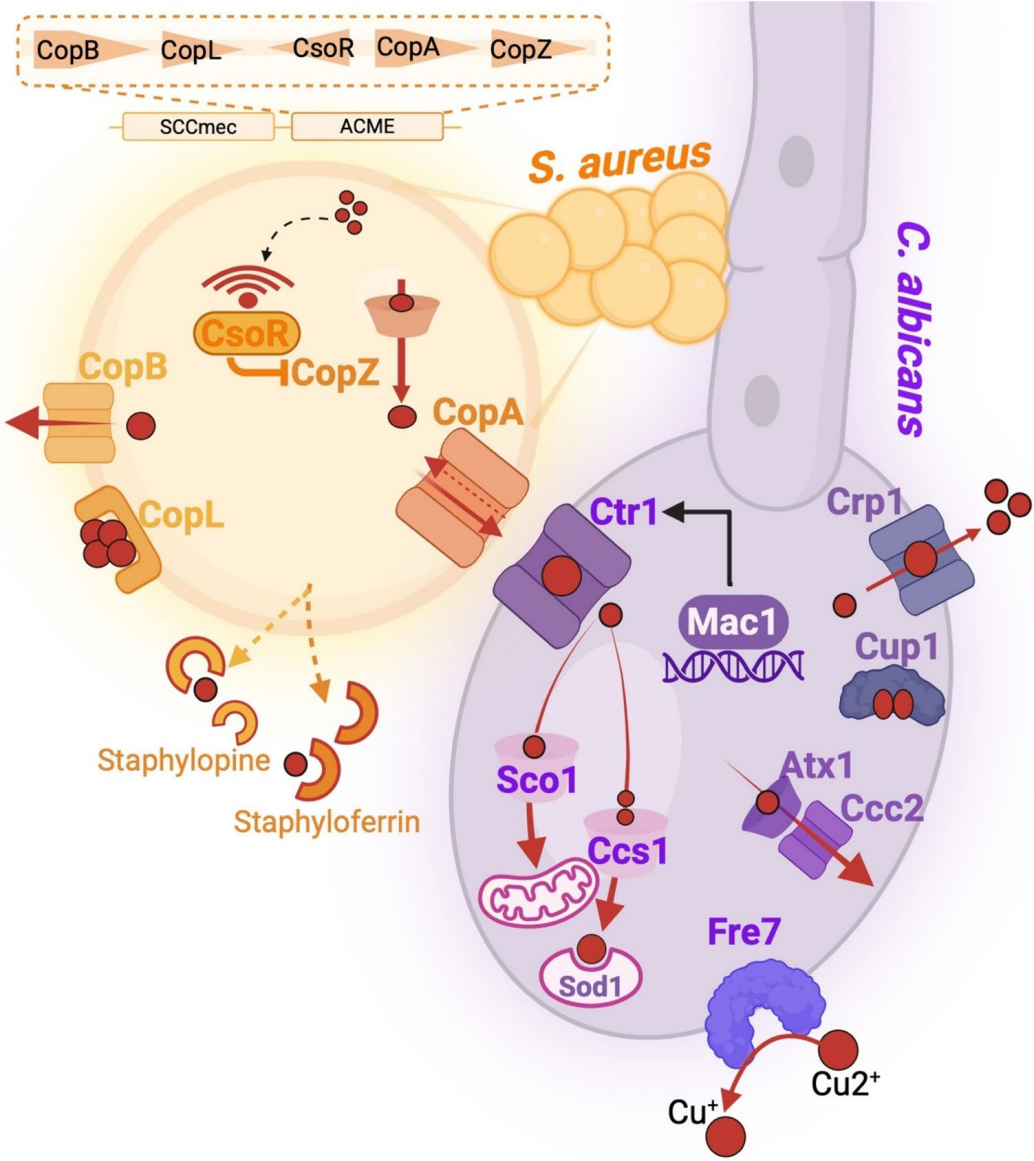
Schematic overview of copper acquisition, transport and detoxification machinery shaping copper availability during *S. aureus*–*C. albicans* interaction. In S*. aureus*, copper stress is sensed by **CsoR**, a Cu(I)-responsive transcriptional repressor that regulates expression of copper-detoxification genes. **CopZ** functions as a cytosolic copper metallochaperone that binds Cu(I) and delivers it to copper-export systems, such as **CopA**, a P-type ATPase that exports excess cytosolic copper to mitigate copper toxicity. Mobile-element-associated copper-resistance determinants include **CopB**, a copper transporter and **CopL**, a copper-binding lipoprotein that contributes to copper sequestration. These determinants are encoded on a locus with SCCmec, linking copper tolerance to staphylococcal resistance. Broader metal-acquisition through the secreted metallophore staphylopine and siderophore staphyloferrin, may influence copper availability in the extracellular shared environment. In *C. albicans*, extracellular Cu(II) can be reduced to Cu(I) by **Fre**-family metalloreductases, enabling uptake through **Ctr1**, a high-affinity plasma-membrane copper transporter. Copper responses are regulated by **Mac1**, a copper-responsive transcription factor that activates copper-acquisition genes, including *ctr1*. Following uptake, intracellular copper is distributed by copper-trafficking proteins including **Atx1**, a cytosolic copper chaperone that delivers Cu(I) to **Ccc2**, a P-type ATPase that transports copper into the secretory pathway. Copper is also directed to mitochondrial and antioxidant pathways through **Sco1**, which supports mitochondrial copper delivery and cytochrome c oxidase assembly, and **Ccs1**, the copper chaperone that metallates **Sod1**, the Cu/Zn superoxide dismutase required for detoxification of superoxide radicals. Excess copper is detoxified through **Cup1**, a copper-binding metallothionein, and **Crp1**, a copper-exporting P-type ATPase. Together, these systems represent a proposed interkingdom copper-handling network in which fungal and bacterial copper handling machinery intersect to influence mixed community fitness, metal stress tolerance and community stability

Copper biology in *C. albicans* is dynamic because the fungus must survive alternating copper excess and deprivation across host sites, and during host health and disease [40]. Classic work established copper transporter 1 Ctr1 as a functional high-affinity copper transporter and copper resistance protein 1 Crp1 as a major copper-efflux ATPase required for copper tolerance (Fig. 3) [36, 45]. During systemic candidiasis, Mackie and colleagues showed that infected kidneys undergo a transient early copper increase followed by later decline; *C. albicans* responds by upregulating *crp1* during early infection and *ctr1* later, and both genes are required for full virulence [40].

Copper is also central to the fungus’ metabolic adaptability: *C. albicans* swaps metal cofactors for cytosolic Sod, using Cu/Zn-Sod1 when copper is available, and Mn-Sod3 when it is scarce [46]. Additionally, copper starvation induces alternative oxidase-dependent respiratory remodelling in *C. albicans* [47]. Transcriptomic studies have demonstrated how copper availability acts as a cue extending beyond metal detoxification, and reshapes antioxidant defence, iron acquisition, metabolism, epithelial adhesion, host-cell damage and antifungal tolerance [48]. Therefore, illustrating how copper exposure within the cuprosphere can shape fungal pathogenic potential.

### Copper & *S. aureus*: Detoxification, Virulence and AMR Risk

In *S. aureus*, our knowledge of copper handling is classically framed around the copper-sensitive operon repressor CsoR and the core homeostasis machinery that includes CopA and CopZ [37, 49]. This core system contributes to pathogenesis, and many clinically important strains carry additional horizontally acquired loci that substantially extend tolerance including CopB-mco, CopXL, and CopBL/CopL (Fig. 3). These loci increase survival under copper intoxication and improve fitness inside macrophages, in human blood, or in animal infection models [50–52].

It is accepted that acquired copper hypertolerance is now part of the virulence toolkit of some *S. aureus* lineages [53]. This is particularly important in livestock-associated and epidemic MRSA. A 2023 UK government review concluded that there is evidence that heavy metals can affect AMR development in food-animal production, that heavy metals persist for years, and that copper resistance genes such as copB can be associated with mobile genetic elements that confer methicillin resistance in *S. aureus* in livestock-associated MRSA, such as SCCmec (Staphylococcal Cassette Chromosome mec) [54, 55]. The report identifies copper used in animal food production as an unresolved One Health AMR risk and highlights the need for better surveillance in animal, aquaculture, manure and soil systems. It argues for longitudinal field and metagenomic studies to determine whether heavy metal exposure drives AMR transmission through animals, soils and the food chain. However, the same report also stressed that direct field evidence tracing this all the way to consumer risk remains insufficient.

### Copper Asymmetry and Interkingdom Synergy

The contrast between the two organisms copper handling is striking. *C. albicans* appears adapted to fluctuating copper availability, shifting between copper import, export and alternative metal cofactor use across host-relevant conditions [40, 47]. *S. aureus*, by contrast, appears especially adapted to surviving copper intoxication, relying on detoxification systems that can be reinforced in some lineages by acquired mobile copper-resistance loci [56]. This tracks with each organism’s different tolerance for copper [36, 37], and this asymmetry of copper tolerance makes division of labour in mixed communities biologically plausible.

The pairing of *C. albicans*–*S. aureus* has long been a compelling model system for studying fungal-bacterial interactions owing to the organisms’ individual significance to human health and disease, their popularity as tractable models for fungal and bacterial pathogenicity respectively, and their intriguing mutualism in many models. During their interaction, *S. aureus* binds specifically to *C. albicans* hyphae through the fungal adhesin Als3 [57], and co-infection studies show enhanced bacterial dissemination through host tissues [58, 59]. In vivo, these organisms result in co-infections significantly worse than their respective mono-infections [60–62]. Fungal regulation of metabolism was shown to underpin infectious synergism by enhancing *S. aureus* toxicity, leading to organ injury and polymicrobial sepsis [63].

Beyond human infection*, S. aureus* is a major bovine mastitis pathogen, causing persistent subclinical and clinical udder infections that reduce milk yield and quality, and are recalcitrant to treatment [64]. Additionally, bovine mastitis has also been associated with high levels of *Candida* species in the milk [65], raising the possibility that resilient communities of these organisms are contributing to bovine mastitis as well as human co-infections [66].

### Copper-Mediated Interkingdom Biofilm Mutualism

Biofilms represent the predominant microbial lifestyle. Aggregates of fungi and bacteria, encased in a protective self-produced matrix, are found in soil, mammalian microbiomes and water systems [67]. Importantly, biofilms provide a natural protection for microbes with elevated MICs, reduced penetration of drugs through matrix, and reduced metabolic activity, or even dormancy, in some biofilm dwelling microbes [68]. Thus, when considering tackling microbial resilience in the face of therapeutic intervention we must consider how biofilms build and sustain their resilience.

The first proteomics investigation into *C. albicans* and *S. aureus* mixed biofim found copper homeostasis regulator metal activated transcription factor 1 (Mac1) was differentially regulated by *C. albicans* during biofilm growth with *S. aureus* [69]. Recently, an important role for copper in how fungi and bacteria interact in biofilms has been revealed. Specifically, our global proteomics approach showed reciprocal regulation of copper-handling proteins in both *C. albicans* and *S. aureus* in mixed biofilms. Fungal copper import was increased, as were copper specific chaperones Sco1 and Ccs1, and Sod1 which required copper as a cofactor. Meanwhile, bacterial copper export and supporting chaperones are increased [39]. These data demonstrate how during mixed biofilm growth, bacterial copper export coincides with increased fungal copper import, supporting a model in which copper availability is redistributed across kingdoms during mixed biofilm growth. Importantly, this is linked to the biomass of the biofilms, and when this copper sharing is disrupted either by altering available copper in the media or genetic deletion of fungal copper import (*C. albicans ctr1* null mutant), mixed biofilms fail to reach biomass of wild type counterparts. These findings support the central argument of this review: fungi and bacteria can use copper to build and sustain resilient interkingdom interactions.

## Knowledge Gaps and Opportunities

Key gaps remain in quantifying how copper shapes fungal–bacterial communities across plant, animal and human-associated environments. Greater epidemiological continuity is needed, particularly studies that trace copper-resistance genes, mobile elements and strain lineages across farm effluent, wastewater, surfaces and clinical infections – ideally, within a single surveillance framework. Copper speciation is also often overlooked: most surveillance measures total copper mass, whereas biological effects depend on ionic state, complexation, redox chemistry and particulate form.

These gaps translate into One Health hypotheses. Copper-rich soils, slurry or wastewater may prime isolates for improved survival under phagocyte copper stress and enhanced persistence in mixed *Candida–Staphylococcus* biofilms. Different copper forms may impose distinct selective pressures, with soluble inorganic copper, metallic copper surfaces and organo-copper formulations differing in environmental loading and resistance mobility. Biofilm architecture may also generate copper gradients that shift host copper stress from a lethal burst toward a buffered community resource.

Together, these observations suggest areas where copper stewardship merits closer scrutiny: chronic background loading, formulation choice, downstream effects on gut, slurry and soil resistomes, and the distinction between high-kill copper surfaces and sublethal exposures. The ultimate aim in understanding these processes is to deploy copper with greater ecological precision.

## Conclusion

Across the cuprosphere, copper can suppress susceptible fungi, enrich for copper-tolerant communities, remodel fungal–bacterial networks and influence resistance-associated traits. In human-associated fungi such as *C. albicans*, copper also acts as a host-imposed stress, a cue for metabolic adaptation and, in mixed biofilms with *S. aureus*, a potential mediator of interkingdom cooperation. Understanding when copper controls fungal growth and when it supports persistence, pathogenicity or polymicrobial resilience will be essential for more precise copper stewardship across agriculture, the environment and infection control.

## Key References

- Pesce S, Mamy L, Sanchez W, et al. (2024) The use of copper as plant protection product contributes to environmental contamination and resulting impacts on terrestrial and aquatic biodiversity and ecosystem functions. Environmental Science and Pollution Research 32:2830–2846. 10.1007/s11356-024-32145-z.
  - Frames agricultural copper use as a persistent environmental contaminant that can reshape biodiversity and microbial selection across diverse ecosystems.
- Hall J, Mekapothula S, Coxhill R, et al. (2024) Surface-Functionalised Copper Oxide Nanoparticles: A Pathway to Multidrug-Resistant Pathogen Control in Medical Devices. Nanomaterials 14:1899. 10.3390/nano14231899.
  - Illustrates the current clinical potential of copper-based surface technologies for controlling multidrug-resistant pathogens on medical devices.
- Wen Y, Gao M, Wang Z, et al. (2026) Dietary copper-driven colonic dysbiosis mediates oxidative stress and butyrate deficiency to facilitate the spread of resistome in pigs. NPJ Biofilms Microbiomes 12:. 10.1038/s41522-026-00949-1.
  - Links dietary copper to resistome expansion, strengthening the One Health case for copper stewardship.
- Schneider M, Deltedesco E, Gorfer M, et al. (2025) Copper-based fungicide application shifts the soil bacterial community structure and the soil nitrogen cycle. Plant Soil 513:1807–1825. 10.1007/s11104-025-07278-w.
  - This study shows how copper fungicide exposure perturbs microbial ecology beyond its intended antifungal target.
- Kalinina I, Vazquez-Muñoz R, Ross O, et al. (2026) Copper-driven mutualism of Candida albicans and Staphylococcus aureus interkingdom biofilms. Microbiology (N Y) 172:. 10.1099/mic.0.001725.
  - This study provides the mechanistic basis and entry point for this review’s central thesis that copper can act as a shared resource that sustains interkingdom interactions.
- Li J, Zheng Q, Liu J, et al. (2024) Bacterial-fungal interactions and response to heavy metal contamination of soil in agricultural areas. Front Microbiol 15:1395154. 10.3389/fmicb.2024.1395154.
  - This study demonstrates how copper-rich environments can reshape interkingdom community structure in soil.
- Lu J, Liu X, Li X, et al. (2024) Copper regulates the host innate immune response against bacterial infection via activation of ALPK1 kinase. Proc Natl Acad Sci U S A 121:e2311630121. 10.1073/pnas.2311630121.
  - This study frames copper as an active immune signalling molecule.
- Solier S, Müller S, Cañeque T, et al. (2023) A druggable copper-signalling pathway that drives inflammation. Nature 617:386–394. 10.1038/s41586-023-06017-4.
  - An elegant work placing copper upstream of inflammatory cell control and demonstrating how copper signalling may be druggable.

## Data Availability

No datasets were generated or analysed during the current study.
